# Soil Respiration and Bacterial Structure and Function after 17 Years of a Reciprocal Soil Transplant Experiment

**DOI:** 10.1371/journal.pone.0150599

**Published:** 2016-03-02

**Authors:** Ben Bond-Lamberty, Harvey Bolton, Sarah Fansler, Alejandro Heredia-Langner, Chongxuan Liu, Lee Ann McCue, Jeffrey Smith, Vanessa Bailey

**Affiliations:** 1 Pacific Northwest National Laboratory, Joint Global Change Research Institute at the University of Maryland–College Park, 5825 University Research Court #3500, College Park, MD, 20740, United States of America; 2 Pacific Northwest National Laboratory, 902 Battelle Boulevard, Richland, WA, 99352, United States of America; 3 USDA-ARS, 215 Johnson Hall, Washington State University, Pullman, WA, 99164, United States of America; UC Irvine, UNITED STATES

## Abstract

The effects of climate change on soil organic matter—its structure, microbial community, carbon storage, and respiration response—remain uncertain and widely debated. In addition, the effects of climate changes on ecosystem structure and function are often modulated or delayed, meaning that short-term experiments are not sufficient to characterize ecosystem responses. This study capitalized on a long-term reciprocal soil transplant experiment to examine the response of dryland soils to climate change. The two transplant sites were separated by 500 m of elevation on the same mountain slope in eastern Washington state, USA, and had similar plant species and soil types. We resampled the original 1994 soil transplants and controls, measuring CO_2_ production, temperature response, enzyme activity, and bacterial community structure after 17 years. Over a laboratory incubation of 100 days, reciprocally transplanted soils respired roughly equal cumulative amounts of carbon as non-transplanted controls from the same site. Soils transplanted from the hot, dry, lower site to the cooler and wetter (difference of -5°C monthly maximum air temperature, +50 mm yr^-1^ precipitation) upper site exhibited almost no respiratory response to temperature (Q_10_ of 1.1), but soils originally from the upper, cooler site had generally higher respiration rates. The bacterial community structure of transplants did not differ significantly from that of untransplanted controls, however. Slight differences in local climate between the upper and lower Rattlesnake locations, simulated with environmental control chambers during the incubation, thus prompted significant differences in microbial activity, with no observed change to bacterial structure. These results support the idea that environmental shifts can influence soil C through metabolic changes, and suggest that microbial populations responsible for soil heterotrophic respiration may be constrained in surprising ways, even as shorter- and longer-term soil microbial dynamics may be significantly different under changing climate.

## Introduction

Understanding how climate change will affect soil carbon (C) cycling is critical for predicting future changes in the ecosystem- to global-scale C cycle [1]. Specifically, the effects of climate change on the structure, microbial community, C storage, and respiration response of soil organic matter (SOM) remain uncertain and widely debated [2–4]. With respect to SOM decomposition, two broad dynamics can result in differences between the short- and longer-term respiratory responses: substrate depletion and acclimation [5]. In the first, soil C pools become depleted under warmer conditions, for example if net primary production (the ultimate source of SOM) is relatively constrained by, e.g., nitrogen limitations [6]. In the second, changes in microbial metabolic pathways or community structure might result in less-efficient decomposition regardless of substrate change [7, 8], limiting any positive feedback effect from SOM decomposition. The relative importance of these two broad mechanisms is debated [3], and global models reflect this uncertainty, exhibiting highly divergent responses to future climate change [9, 10].

Uncertainties are particularly high in dryland ecosystems, in which small changes in precipitation, in particular with respect to infrequent precipitation events, can strongly influence biological activity and C cycling [11, 12] with global effects [13]. Microbial community responses to changes in abiotic drivers, especially precipitation, are widely divergent (recently summarized by [11]), with most based on relatively short-term field and/or laboratory manipulative experiments. But the effects of climate changes on ecosystem structure and function are often modulated or delayed [14]. Long-term manipulative experiments include both active and passive warming designs, the longest of which have run for one to two decades [15, 16]. Many shorter-term experiments have also manipulated soil and/or air temperatures to study warming effects on ecosystem processes [17], but such experiments may not accurately predict longer-term changes as ecosystems respond to complex climate shifts [18–20].

Transplant studies between different elevations or latitudes provide an alternative to *in situ* manipulation. Reciprocal transplants, in which climate and other factors (e.g. vegetation type) vary, have examined areas such as microbial community structure (e.g., [21]), litter decomposition [22, 23], soil respiration [24], and nitrogen dynamics [25]. In a Washington state (U.S.) dryland, Link et al. [26] examined the effects of climate change (as expressed via a reciprocal transplant experiment) on grassland plants and soils, including shoot C isotope discrimination, plant density, and SOM fractions. Such changes are important to understand in arid areas, which are considered particularly vulnerable to climate changes [27].

This study examined the response of dryland soils using a long-term (17 year) experiment, first reported by Link et al. [26], in which soil cores were transplanted between and within two sites on a mountain in eastern Washington, USA. The two sites’ similar soil types, and multiple experimental controls, provided a strong test of the longer-term effects of climate change. Based on a key finding of Link et al. [26], who observed a rapid decrease in active-fraction soil C under hotter, drier conditions, we hypothesized that continued changes in soil structure and biogeochemical dynamics would lead to shifts in the structure and function of the soil biota. This in turn would be expected to produce differences in SOM decomposition and soil respiration between the experimental treatments, providing a feedback mechanism between climate change effects on soils and atmospheric C.

We conducted a laboratory incubation to examine the respiratory potential and differences in the biological responses of these soils after the 17-year transplant. Carbon dioxide (CO_2_) evolution was measured, enzymatic and microbial assays performed, and the bacterial community profiled to examine how almost two decades of exposure to local climate had affected the functionality of these soils. Such an integrative approach has been called for in several recent perspectives on soil and climate change [3, 20, 28].

## Methods

### Study sites

This study examined soils originating from two different-elevation sites at the Fitzner-Eberhart Arid Lands Ecology Reserve on Rattlesnake Mountain (46.406°N, 119.611°W) located in semiarid southeastern Washington, USA. The sites and mountain are part of the Hanford Reach National Monument, and generally considered pristine. All permits and policies for this experiment were compliant with the U.S. Department of Energy NEPA (National Environmental Policy Act). No protected species were sampled.

The sites had different elevations and climates but similar plant communities and soil types (**Table 1**). The lower elevation (310 m) site is warmer (28.5°C air average monthly maximum) and drier (224 mm yr^-1^) than the upper elevation site (844 m, 23.5°C, 272 mm yr^-1^ respectively). The sites have ~2% slopes, with north (lower) and northeast (upper) aspects respectively. Both soils are silt loams (coarse-silty, mixed, mesic Xerollic Camborthids) derived from the same basalt loess parent material, with identical mineralogy [29]. The plant community of the lower site is dominated by *Pseudoroegneria spicata* (Pursh) Á Löve and *Poa secunda*, while the upper site is dominated by *Artemisia tripartita* Rydb., *P*. *spicata*, and *P*. *secunda* [26].

**Table 1:** Environmental and soil characteristics of the upper and lower sites on Rattlesnake Mountain, Washington, USA. Environmental data include long-term mean annual temperature (MAT) and precipitation (MAP) based on both older [63] and recent (unpublished weather station data) sources; this climatic regime has been broadly stable for the last 3000–5000 years [64]. Soil values are 0–5 cm means±s.d. of the ‘native’ cores sampled from upper and lower sites (N = 24). Bulk density, carbon and nitrogen, particulate organic matter (POM) C, and POM N all differed significantly (P<0.001) between the lower and upper sites. Soil cores were taken from areas of the two sites dominated by *Poa* spp.

	Upper site	Lower site
Altitude (m a.s.l.)	844	310
MAT (°C)	23.5	28.5
MAP (mm)	272	224
Plant species	*Poa secunda*, *Pseudoroegneria spicata*	*P*. *spicata*, *P*. *secunda*, *Artmesia tripartita*
Soils	Silt loams (coarse-silty, mixed, mesic Xerollic Camborthids)	
Bulk density (g cm^-3^)	1.42±0.07	1.51±0.08
Carbon (0–5 cm, %)	2.04±0.81	0.95±0.32
Nitrogen (0–5 cm, %)	0.19±0.08	0.09±0.03
POM C (%)	0.27±0.09	0.07±0.03
POM N (%)	3.00±1.02	0.89±0.35
C:N (0–5 cm)	10.5	10.1

In 1994, a reciprocal transplant experiment was conducted between these two sites, with thirty-one 30.5-cm diameter, 30-cm deep soil cores reciprocally transplanted between the top and bottom site; separate cores were also transplanted within sites to control for disturbance effects. The PVC tubes used to transplant the cores remained in place as a physical barrier, so the soil cores were in contact with the surrounding soil only through the bottom of the 30-cm tube. This experiment was designed to test whether soil C and N would decrease in a warmer, drier climate and increase in a cooler, wetter one. Early results (after 4–5 years) supported this hypothesis [26].

We revisited these sites in March 2012. The original cores—both transplants, (moved between elevations) and controls (moved within each elevation)—were resampled to investigate the longer-term consequences of this transplant to microbial community composition, soil C and N dynamics, and soil physical structure. We also sampled undisturbed native soils adjacent to, but not part of, the 1994 cores. Our 2012 cores were 3.1 cm wide and 15 cm deep; at each site three were taken from four random 1994 transplant cores, three from four 1994 control cores, and three from native soils. In total we thus took 3 x (4 + 4 + 4) x 2 sites = 72 cores. We exclusively sampled from *Poa*-dominated areas of the sites [26].

### Measurements and incubation

The resampled soils were randomly assigned to one of three experimental groups: (1) destructive time zero analyses; (2) incubation in conditions simulating the hotter, drier lower site; and (3) incubation in conditions simulating the cooler, moister upper site. Eighteen of the 24 total cores in group 1 were analyzed for total C, total N, particulate organic matter (POM)-C and N [30], ß-glucosidase (EC 3.2.1.21) and N-acetylglucosaminidase (EC 3.2.1.30) activity using methylumbelliferone-labeled model substrates, and bacterial community structure. Six group 1 cores were set aside for physical characterization of core porosity using X-ray microtomography and root sampling; these results were not part of the experiment described here, but are discussed by Yang et al. [31]. The chemical and biological assays were conducted for three soil depths (0–5, 5–10, and 10–15 cm, separated in the lab) and all replicates.

**Fig 1:**
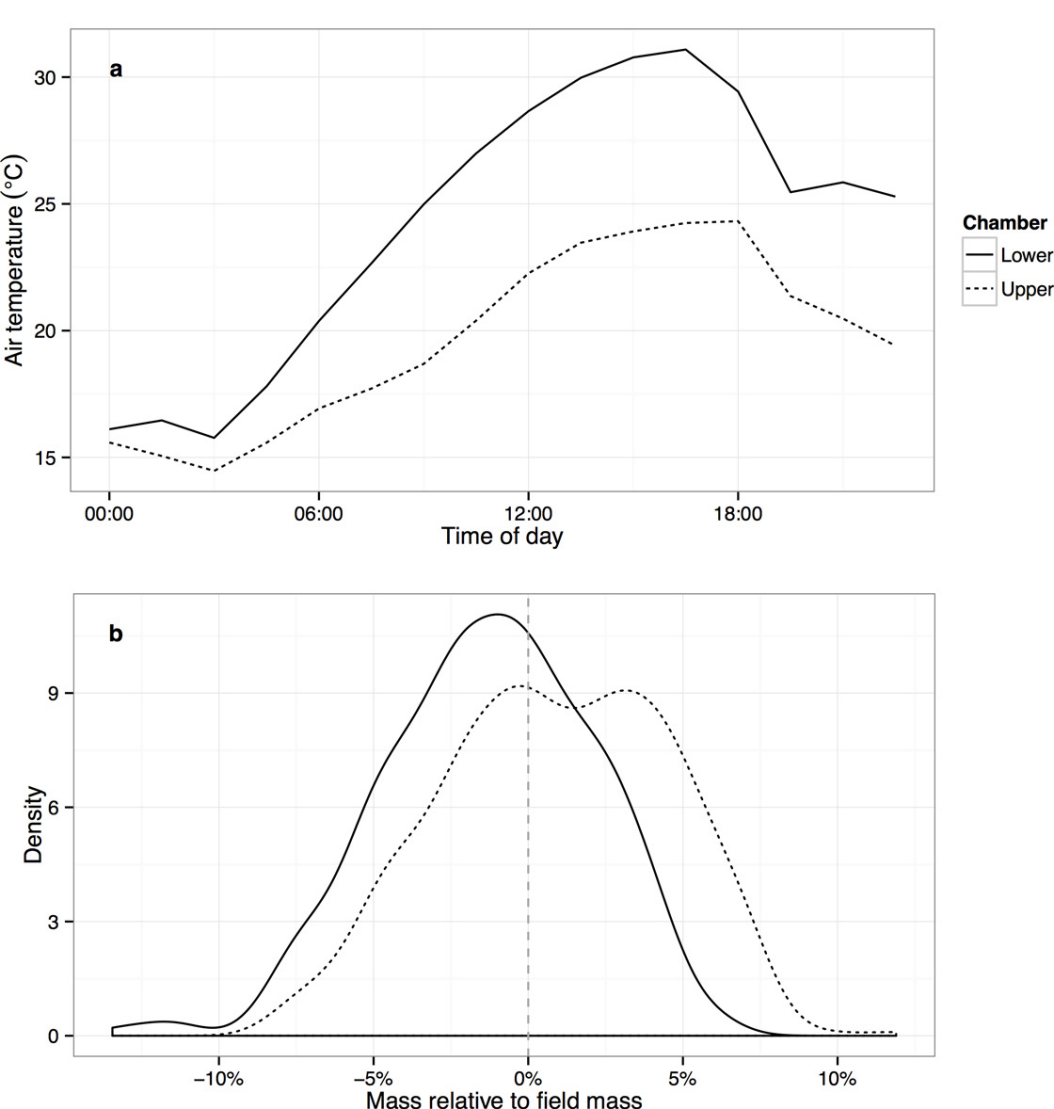
**Daily air temperature cycle (a) and density plot (showing normalized distribution of data) of core soil moisture status (b), by incubation chamber, over the 100-day incubation.** The two chambers mimicked the lower and upper sites, respectively, on Rattlesnake Mountain.

We found that four cores in groups 2 and 3 were damaged, and removed them from the experiment. The remaining 44 cores were incubated for 100 days, in an experiment designed to assess the cores’ respiration response to temperature throughout a realistic daily temperature and light cycle. Cores were randomly assigned to one of two growth chambers (Conviron BDW80, Winnipeg, Canada) simulating early-summer conditions in air temperature, relative humidity, and photosynthetic photon flux density (PPFD), matching data reported by meteorological stations at similar elevations and aspects on the same mountain. One chamber simulated the cooler, wetter upper site (daily cycle of 14.5–24.3°C, RH 25–46%, PPFD 0–1050 μmol m^-2^ s^-1^) and the other hotter, drier lower site (15.8–31.1°C, RH 21–49%, PPFD 0–987 μmol m^-2^ s^-1^) (**Fig 1**). The cores were mounted on 0.05-MPa ceramic plates (Soil Moisture Equipment Corp., Santa Barbara, CA) so that, when the plates were placed in contact with water, water would move up into the cores via capillary action. As this experiment was designed to focus on temperature response, we attempted to keep the cores within ±5% of their field capacity (~27%) throughout the experiment, placing the pore plates in contact with water when necessary.

During the incubation, respiration measurements were made from all cores using an EGM-4 infrared gas analyzer (IRGA; PP-Systems, Amesbury, MA) connected to a custom PVC cap that fit snugly over the cores. We measured respiration approximately every week, at a variety of air temperatures (i.e., times in the diurnal cycle), and in a random order. After the cap was put in place, a core was allowed to equilibrate for 10–20 seconds before measurements began (manually controlled; typically six CO_2_ concentration readings were taken within 30 seconds, with air temperature logged simultaneously). Core mass fluctuated with water content, and was recorded after each measurement. One core began to fragment when moved, and to prevent further disturbance to the soil matrix and the respiration measurements, its day-to-day mass was instead computed based on the relative mass changes of the other cores in the same chamber. At the end of the incubation period, the same time-zero analyses described above were conducted on all incubated cores, sliced into 5 cm increments. Most notably, bacterial community structure was again examined with targeted sequencing of the V4 region of the 16S rRNA gene as described below.

### Data analysis

The 4–8 individual IRGA CO_2_ readings for each core and measurement were examined for obvious outliers and then a linear rate of change (*δc/δt*) for CO_2_ concentration was computed. Each core’s respiration flux (*F*) was then calculated following e.g. Steduto et al. [32] as

$$F=\frac{\delta c}{\delta t}\frac{V}{M}\frac{Pa}{RT}$$

where *V* is the core-specific system volume, *M* the core dry mass as determined at the end of the incubation, *P*_*a*_ atmospheric pressure (101 kPa; the incubation chambers were ~120 m a.s.l.), *R* the universal gas constant (8.3 x 10^−3^ m^3^ kPa mol^-1^ K^-1^) and *T* the chamber air temperature (K) at time of measurement. The final respiration rate was expressed as mg C kg soil^-1^ day^-1^. All analyses were performed using the R language for statistical computing [33] version 3.0.2.

We used a linear model (*lm* in R) to test the fixed effects of core source (i.e., the site from which a core originally came), location (where it spent 1994–2012), and type (transplant versus native) on respiration rate (log *F* above; we transformed the dependent variable to allow for a nonlinear response). Core bulk density, water content, and incubation day were all tested for their effects on *F*. Air temperature for each observation was normalized relative to the chamber mean, resulting in adjusted *T* values of similar range for each chamber and thus facilitating their comparison [34]. Time-zero analyses were tested using multi-way analysis of variance in R, testing both individual treatments effects and their first-order interactions.

The basal respiration and temperature sensitivity of the bulk respiration data were estimated with a Q_10_-style function [35] using nonlinear least squares (*nls*) in R, i.e.

$$F=F_{20}{Q_{10}}^{(T-20)/10}$$

where *F* is as above, *F*_20_ is the flux (respiration rate) at 20°C, *T* the chamber air temperature (here°C), and *Q*_10_ the ‘apparent’ [36] temperature sensitivity. The algorithm used initial-guess values of 5 mg C kg soil^-1^ day^-1^ for *F*_20_ and 2.0 for *Q*_10_. This equation is an empirical convenience [36, 37] but one that fit these data well with no trend or heteroscedasticity in its residuals (data not shown).

DNA was extracted from 0.25 g of soil per sample using the PowerSoil® DNA Isolation Kit (http://www.mobio.com/) according to the manufacturer’s instructions. PCR amplification of the V4 region of the 16S rRNA gene was performed using the protocol developed by the Earth Microbiome Project (http://press.igsb.anl.gov/earthmicrobiome/emp-standard-protocols/16s/), and described in Caporaso et al. [38], with the exception that the twelve base barcode sequence was included in the forward primer. Amplicons were sequenced on an Illumina MiSeq using the 500 cycle MiSeq Reagent Kit v2 (http://www.illumina.com/) according to the manufacturer’s instructions.

We used the 16S sequence data to compare the bacterial community structures of all soil samples. The sequence data were demultiplexed and the paired ends joined, requiring an overlap of 100 bases with < 5% difference, using ea-utils (v.1.1.2–537; https://code.google.com/p/ea-utils/). High quality joined sequences were converted from fastq to fasta using BioPerl (www.bioperl.org) and processed using *mothur* v.1.30.1 [39]. Briefly, sequences with ambiguous bases were excluded, as were sequences that: (1) did not align to the V4 region of the Silva 16S rDNA reference alignment (http://www.arb-silva.de/) [40], or (2) were identified as chimeric by both UCHIME (http://drive5.com/uchime/) [41] and ChimeraSlayer (http://microbiomeutil.sourceforge.net/#A_CS) [42], or (3) were classified as chloroplast, mitochondria, or unclassified by the RDP reference taxonomy (http://rdp.cme.msu.edu/index.jsp) [43]. After this processing, five samples yielded no sequences, and the remaining 193 samples (3 depth intervals x 3–4 replicates, depending on group) yielded 4,426,880 sequences (~23,000 per sample on average). Randomly subsampling 10,000 sequences from each sample eliminated five additional samples with insufficient data; the remaining 188 samples were retained for analysis. The 1,880,000 sequences were assigned to OTUs at ≥ 97% identity (with furthest neighbor linkage), and taxonomy assigned using the RDP reference taxonomy. Non-metric multidimensional scaling was performed in *mothur* (“nmds” command), using the Morisita-Horn index to describe the dissimilarity in community structure between samples, and the resulting ordination visualized in MATLAB® (MathWorks, Inc.). Analysis of molecular variance (AMOVA in *mothur*) was used to test transplant location and incubation effects on the community structure.

## Results

Native soils differed significantly between the upper and lower Rattlesnake Mountain sites (**Table 1**), with the lower site having higher bulk density and lower C and N, consistent with it being a hotter and drier environment. Transplantation did not generally have any effect on soil structural and chemical properties, however: the site at which cores spent the years 1994–2012 did not significantly affect the bulk density of the 15-cm soil cores (*P* = 0.119), 0–5 cm percent C (*P* = 0.834), 0–5 cm percent N (*P* = 0.569), or any of the other structural and chemical properties measured.

Cores were generally maintained within ±5% of their field water content (i.e., the level at the time of sampling), and almost always within ±10% (Fig 1). Native soils from the lower site exhibited higher water content variability than all other samples: overall native soils water content was 28.4% ± 7.5% (mean ± s.d.), and transplanted soils 27.6% ± 5.4%; lower source cores 25.7% ± 4.5%, upper source cores 29.3% ± 5.6% (S1 Fig). This suggests that the lower organic matter content of the lower site was associated with lower water holding capacity.

**Fig 2:**
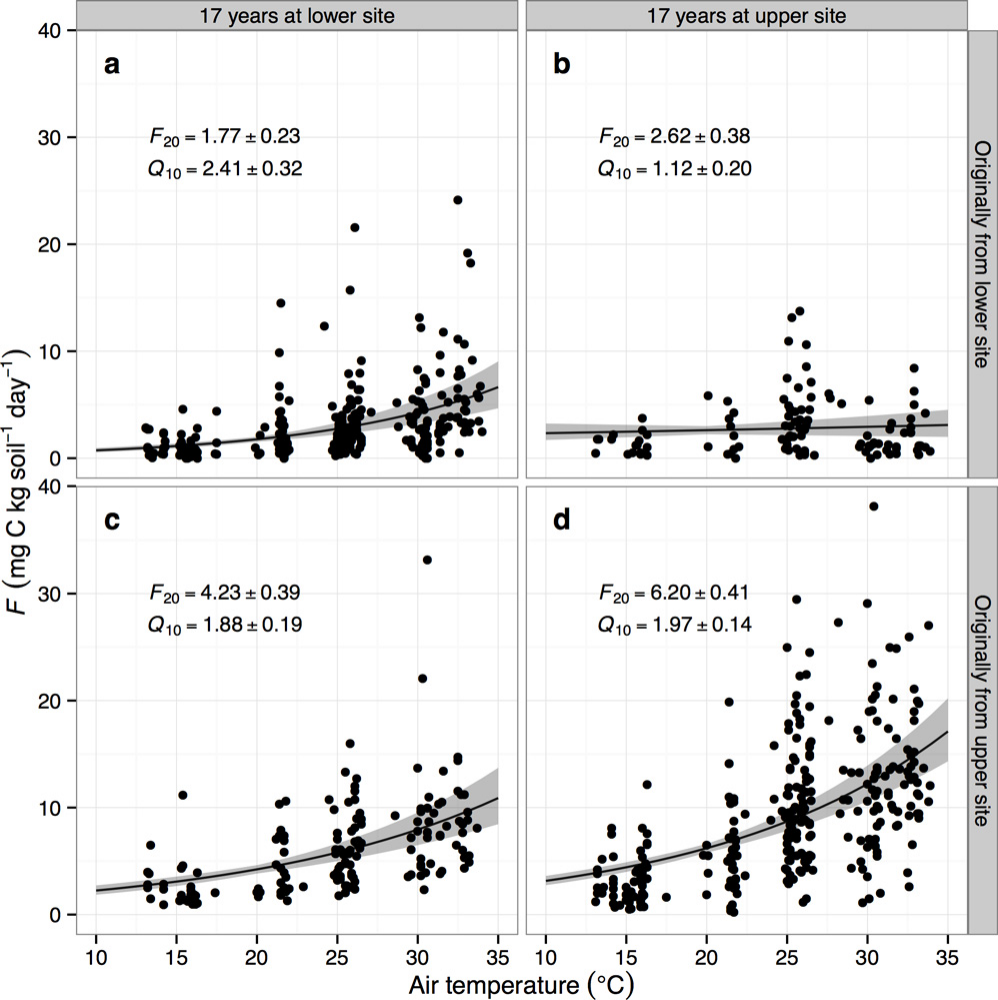
Soil respiration (*F*) as a function of temperature, by core origin and location over the 17-year transplant experiment. Cores originally from the lower site (panels a, b) respired less than those originally from the upper site (c, d). Values for respiration at 20°C (*F*_20_, mg C kg soil^-1^ day^-1^) and *Q*_10_ are also given with SEs, based on combined data from both incubation treatments. Curves and shaded error regions are the Q_10_-style models with parameters given in each panel.

The linear-effects model used to analyze the incubation experiment data is summarized in Table 2. All cores originally from the upper, cooler site had significantly higher respiration rate at 20°C (*F*_20_) than those from the lower site (*P*<0.001). Within-site transplanted cores (i.e. upper-to-upper and lower-to-lower controls, transplanted in 1994) respired more (*P*<0.001) than the native controls, suggesting a significant disturbance effect still present after almost two decades. Air temperature and soil water content were significantly (*P*<0.001 for both, Table 2) and positively correlated with *F*. Cores originally from the lower site that spent 17 years at the upper site had very little temperature sensitivity (*Q*_10_ = 1.12; Fig 2); this effect was highly significant (*P* = 0.010). Neither incubation day (i.e. time) nor bulk density was significant in any analysis. The overall respiratory *Q*_10_ response—the soils’ apparent [36] temperature sensitivity, across all treatments—was 2.42±0.32 with a mean basal respiration rate at 20°C (*F*_20_) of 3.95±0.23 mg C kg soil^-1^ day^-1^.

**Table 2:** Summary of the linear model of soil core respiration. Terms include parameter estimate based on type III sum of squares, standard error (SE), t-value, and *P*-values. Effects include core location (1994–2012 experiment, Upper or Lower), core source (pre-1994 location, Upper or Lower site; cf. Table 1), core type (Native or Transplant), water content (WC, percent, gravimetric), relative air temperature (T_rel_, i.e. normalized against each incubation chamber’s mean temperature), and chamber (simulating conditions for the Upper or Lower site). A colon denotes an interaction between the main effects above. For example, the “LocationUpper” value means that cores that spent 17 years at the upper site exhibited a flux rate 0.311 log(mg C kg^-1^ soil day^-1^) lower than cores transplanted to the lower site, after all other factors are considered. The dependent model variable *F* has units of mg C kg^-1^ soil day^-1^ and was log-transformed prior to modeling. The model had an overall Akaike’s Information Criterion of 1809 and Schwarz's Bayesian criterion of 1861.

Parameter	Estimate	SE	t	*P*
(Intercept)	-1.094	0.139	-7.876	<0.001
LocationUpper	-0.311	0.098	-3.175	0.002
SourceUpper	0.971	0.089	10.938	<0.001
Transplant	0.322	0.057	5.684	<0.001
WC	6.221	0.419	14.852	<0.001
T_rel_	0.099	0.005	21.183	<0.001
ChamberUpper	-0.176	0.075	-2.346	0.019
LocationUpper:ChamberUpper	0.412	0.102	4.053	<0.001
LocationUpper:SourceUpper	0.677	0.117	5.761	<0.001
SourceUpper:ChamberUpper	-0.716	0.106	-6.764	<0.001

The net result for the entire incubation period was that the lower site control cores respired 0.041 g C (normalized per kg C soil: 0.44 g kg^-1^), and the upper control cores 0.079 g C (1.45 g kg^-1^), in the chambers simulating their respective site conditions. The upper-to-lower transplant group respired 0.074 g C (1.05 g mg^-1^) over the 100-day incubation. Finally, the lower-to-upper group respired 0.041 g C (0.55 g mg^-1^), with a higher basal rate (*F*_20_) compensating for the lack of temperature response (Fig 2B). In summary, the transplant effect on total CO_2_ fluxes was significant and reciprocal (Tukey’s HSD difference between upper transplant control and upper-to-lower group *P*<0.001, lower transplant control and lower-to-upper group *P*<0.001): soils originally from the upper site consistently respired more than soils from the lower site.

While CO_2_ fluxes provide a measurement of the current functional responses of the soil, soil enzyme assays provide insights into its physiological potential. For this reason the activities of two broad-acting soil enzymes, β-glucosidase (EC 3.2.1.21) and N-acetyl-β-D-glucosaminidase (EC 3.2.1.30), were measured to assess the general rates of soil microbial activities. Soil depth exerted a significant effect on enzyme activity for both enzymes (both *P*<0.001); the surface 5 cm had the highest activity, with rates decreasing by 15–60% for the 5–10 and >10 cm depths (Table 3). Soils from the two sites were significantly different (*P*<0.001 and *P* = 0.006 for β-glucosidase and NAGase respectively), but generally transplanting had no effect on these soil enzymes below 5 cm.

**Table 3:** Activities of β-glucosidase and N-acetyl-β-D-glucosaminidase in soils from the Rattlesnake Mountain transplant experiment. Soil codes are lower site native (LN), lower control (LC), lower-to-upper transplant (LU), upper native (UN), upper control (UC), and upper-to-lower transplant (UL). Significant differences (labeled as “a”, “b”, etc.) between soils within depths (based on Turkey’s HSD) were only detected in the 0–5 cm depths; values followed by the same letter are not significantly different. Units are μmol MUB g^-1^ soil h^-1^ for both enzymes.

	Soil					
Depth	LN	LC	LU	UN	UC	UL
*β-glucosidase*						
0–5 cm	1.14a	1.62ab	2.48b	2.18b	1.90ab	2.60b
5–10 cm	1.04	0.93	0.99	1.47	1.37	1.24
10 cm-bottom	0.89	0.93	0.91	1.14	1.15	1.00
*N-acetyl-β-D-glucosaminidase*						
0–5 cm	0.79a	0.90ac	1.10ad	1.23bcd	1.02a	1.33bd
5–10 cm	0.76	0.92	0.79	0.95	0.83	0.91
10 cm-bottom	0.75	0.86	0.75	0.92	0.80	0.83

**Fig 3:**
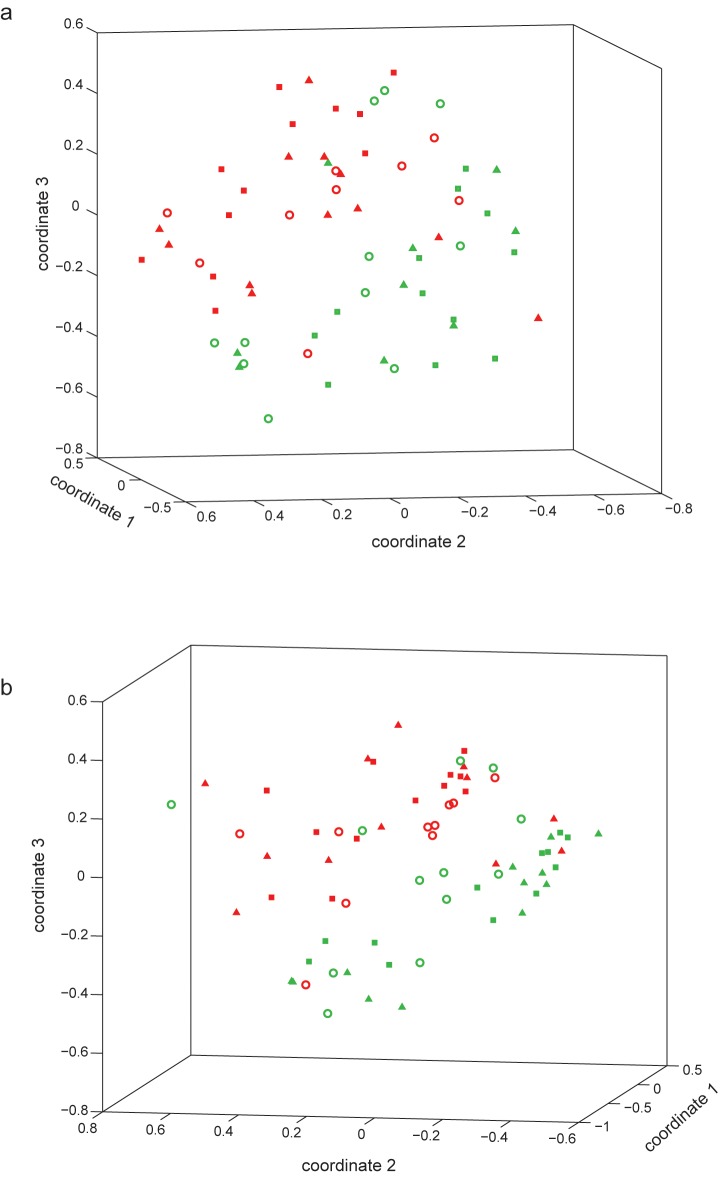
Non-metric multidimensional scaling plot of Morisita-Horn indices (showing dissimilarity in bacterial community structure) for the incubated soils. Red markers indicate soils whose pre-1994 location was the lower site (cf. Table 1), and green markers the upper site. Squares are native soils, triangles are within-site transplant controls, and open circles are the reciprocally transplanted soils. Thus the red open circles represent the soils transplanted from the lower to the upper site, and the green open circles represent the soils transplanted from the upper to the lower site. Soils were incubated in environmental chambers simulating (a) lower or (b) upper site conditions.

Microbial community structure, as measured by 16S sequence sampling, was significantly different between the beginning and end of the incubation experiment, regardless of the chamber in which the cores were incubated (S2 Fig). Soils from the two source locations were significantly different (AMOVA *P* = 0.001 and *P* = 0.002, for lower- and upper-chamber incubated soils, respectively); NMDS plots (Fig 3) of the bacterial community structure of these soils show that the reciprocally transplanted soils grouped with the native and transplant control soils from their pre-transplant location (AMOVA *P*<0.001 and *P* = 0.002 for lower- and upper-chamber incubated soils, respectively). This suggests that the dominant bacterial structure has not changed after the 1994 transplant, even after 17 years.

## Discussion

Several of our results are consistent with previous elevation transplant experiments, although there have been relatively few that examined soil microbial dynamics and/or CO_2_ evolution. Conant et al. [44] reported an 18-month transplant in which soils moved upslope to cooler, mesic sites (very similar to our lower-to-upper transplant reported here) exhibited higher soil water content and soil respiration. Mills et al. [24] performed a multi-site alpine soil transplant to examine climate change effects, and reported that down-slope transplants experienced reduced soil moisture and increased temperature, and reductions in soil respiration, compared to the 1350 m source site. Our results are largely consistent with these studies’ findings. Sjögersten and Wookey [45] measured transplanted cores between low-elevation forest and high-elevation tundra, measuring *in situ* CO_2_ evolution for several years and finding higher rates at the former site. Finally, Hart [46] showed that transplanting in semiarid systems decreased soil microbial biomass size, but increased its activity, and observed that even minor increases in mean annual temperature could have significant impacts on soil N cycling, soil-atmosphere gas fluxes, and soil microbial communities. A common theme to all results was that even small changes in temperature and moisture could have large impacts on soil C and N cycling and microbial communities.

### Functional changes but structural continuity

Two unusual results emerge from this experiment, especially when considered together. First, in the 100-day incubation, while bulk respiration *F* generally varied with both air temperature and soil moisture (even though we attempted to minimize soil moisture variability), it was almost completely temperature-insensitive for lower-site soils transplanted to the upper site (Fig 2B). This suggests a shift in microbial dynamics [8], rather than substrate depletion, since the transplant controls (i.e., lower-to-lower cores) exhibited no such behavior.

The lower site also had significantly lower *F* in general. Soil respiration rates tend to be low in drylands, and the *F*_20_ rates observed here (2–6 mg C kg^-1^ soil day^-1^ or ~8–13 μg C g C^-1^ hr^-1^) were at the lower end of the range observed in an incubation of soils from across North America [47]. While temperature sensitivity of decomposition processes has been widely linked with substrate quality (or organic matter stability), the absence of labile C has also been suggested as a factor in decreasing temperature sensitivity of soil respiration [48]. Curiel Yuste et al. [48] proposed that because labile C depletion occurs more slowly at cooler temperatures, microbial soil respiration would up-regulate or acclimate under such conditions; our results (both cooler-to-warmer and warmer-to-cooler cores, which decreased and increased, respectively, their *F*_20_ values) are consistent with this idea.

Second, both the soil enzymatic (Table 3) and DNA sequence (Fig 3) results suggested that the dominant bacterial membership has not changed in these soils, even 17 years after the 1994 transplant. This is surprising, given the shift in dynamics noted above; much recent work has emphasized that microbial physiology and community structure are critical to understanding soil organic matter dynamics [20, 49], and that changes in community composition are linked to changes in process rates [50]. Taken together, however, our results show that even after 17 years of relocation, the microbial community structure is consistent with controls sampled from the original location, but also that microbial dynamics have significantly changed. In addition, although the respiration analysis (Table 2) showed evidence of a long-standing disturbance effect from the transplant, the within-site transplant controls grouped primarily with the local native soil in the 16S analysis (Fig 3). Thus we consider the overall perturbation (i.e., the 1994 transplant) a “press” perturbation from the years of changed climate rather than a “pulse” perturbation arising from the transplant disturbance [51].

It is intriguing that the differences in local climate between the upper and lower Rattlesnake locations prompt such marked differences in microbial activity (specifically temperature response, Fig 2) in the 100-day incubation with no observed change to bacterial structure after the 17-year transplant (Fig 3). The respiratory insensitivity of the lower-to-upper soils suggests that the microbial community at this site, or its habitat, has been altered such that the community may now lack the functional redundancy or metabolic plasticity [50] needed to adapt to the new climate conditions. Although soil is widely regarded as one of the most functionally and taxonomically diverse microbial habitats on earth [52], highly stressed environments can limit local diversity and presumably functional robustness [53], a situation we propose is occurring in the Rattlesnake Mountain soils studied here. In arid ecosystems in particular, soil heat loads [53] and precipitation patterns [54] may be important controls on soil bacterial and fungal community structure [55]. This may have significant longer-term consequences for the aboveground community [56], and an interesting follow-up study to Link et al. [26] would re-examine the aboveground biota’s structure and function at these Rattlesnake Mountain sites.

### Weaknesses and potential confounding factors

There are a number of caveats to our experimental design that should be noted. Although this experiment reports changes in microbial functionality based on two separate lines of evidence (the bulk CO_2_ respiration data, and β-glucosidase activity) it does not provide a comprehensive evaluation of the soil C cycle (e.g., potential changes in plant-derived C substrates). In addition, the disturbance of core sampling may have broken roots, leaving behind deposits of organic matter that change the water retention capacity of these soils. Substrate depletion could also have occurred because of the slow growth of plants after the 1994 transplant shock, but as noted above the within-site transplant controls showed few differences from the native controls. In addition, soils from the cooler, wetter upper site respired about twice as much C as those from the lower site, and continued respiring almost the same amount (0.074 versus 0.079 g C over 100 days) after 17 years at the hotter, dryer lower site. We would expect this to mean a loss of soil C, consistent with early results from this experiment [26], although we did not observe any significant effect; we measured 2.11±0.49% C in the upper-to-lower cores, compared to 1.36% in the earlier study [26]. Limited root data suggested that upper-site soils had higher root biomass in both native and transplant cores, but we did not have enough samples for statistically valid tests.

Finally, our results are based on the 100-day incubation length used, but temperature sensitivity in particular can vary with incubation time. Wei et al. [7], for example, observed thermal acclimation, with SOM decomposition less temperature-sensitive for soils incubated at higher temperatures; these soils also exhibited microbial community structure (PLFA) shifts. Similarly, Craine et al. [5] found reduced temperature sensitivity with longer incubation periods, suggesting acclimation of some sort by the microbial community. Our temperature sensitivity results are also based on incubation measurements with varying temperatures–i.e., the diurnal cycle simulated by the chambers–in contrast to the more common practice of using a series of controlled, constant temperatures [57]. (Although we note that the approach used here does provide a more realistic temperature environment.) In summary, there are a number of factors in the experimental design and results that mean we cannot provide a definitive picture of all the carbon cycle changes that have occurred during the transplant experiment.

### Conclusions

This is, as far as we know, the longest soil transplant experiment ever reported. Decadal-length experiments [14] have shown that short- and long-term plant production responses may differ significantly, with initial responses modulated or even completely reversed by slower [15] changes in soil biogeochemical cycling. Even a decade was found to be insufficient, however, to observe microbial community changes in colder climates [58], and arid ecosystems may react just as slowly from an experimental perspective. Short-term experiments are thus not sufficient to characterize plant and soil responses, emphasizing the need for further longer-term, multi-factor, and integrative experiments [3].

These results support the idea that environmental change influences soil C cycling through metabolic changes [3, 59, 60]. The stability of the bacterial community structure, in conjunction with the strongly altered CO_2_ flux dynamics, suggest that simulated climate change prompted shifts in either the microbial functionality, which then altered the structure and accessibility of organic matter, or the nature of the soil organic matter, which then forced a shift in the microbial functionality, or both. The modeling implications are potentially significant, as current ecosystem models generally do not incorporate microbial dynamics [61, 62]. This is a particularly acute set of issues for arid and semi-arid ecosystems, which are considered potentially fragile in the face of a changing climate [27], and deserve further study.

## Supporting Information

S1 Fig Gravimetric water content of incubated cores, by core origin and location over the 17-year transplant experiment. Each point shows a single measurement of a single core. Data are shown by core type (native or transplant). Lines around points show distribution of data.
(DOCX)

S2 Fig Non-metric multidimensional scaling plot of Morisita-Horn indices of dissimilarity in bacterial community structure comparing the non-incubated and incubated soils. Data show the 16S bacterial community structure from soil cores. Incubation had a significant effect on the bacterial community structure, with community structure in soils incubated in the lower environmental chamber (green) or the upper environmental chamber (red) clustering separately from that in non-incubated control soils (blue); *P* < 10^−5^.
(DOCX)
