# Supplementary material for: Soil Respiration and Bacterial Structure and Function after 17 Years of a Reciprocal Soil Transplant Experiment
Source: PLoS One. 2016 Mar 2;11(3):e0150599. doi: 10.1371/journal.pone.0150599 (PMC4775055; doi:10.1371/journal.pone.0150599)
Supplement: S1 Fig — Each point shows a single measurement of a single core. Data are shown by core type (native or transplant). Lines around points show distribution of data. (DOCX) [file pone.0150599.s001.docx]

**S1 Fig. Gravimetric water content of incubated cores, by core origin and location over the 17-year transplant experiment.** Each point shows a single measurement of a single core. Data are shown by core type (native or transplant). Lines around points show distribution of data.
