# Supplementary material for: Soil Respiration and Bacterial Structure and Function after 17 Years of a Reciprocal Soil Transplant Experiment
Source: PLoS One. 2016 Mar 2;11(3):e0150599. doi: 10.1371/journal.pone.0150599 (PMC4775055; doi:10.1371/journal.pone.0150599)
Supplement: S2 Fig — Data show the 16S bacterial community structure from soil cores. Incubation had a significant effect on the bacterial community structure, with community structure in soils incubated in the lower environmental chamber (green) or the upper environmental chamber (red) clustering separately from that in non-incubated control soils (blue); P < 10−5. (DOCX) [file pone.0150599.s002.docx]

**S2 Fig. Non-metric multidimensional scaling plot of Morisita-Horn indices of dissimilarity in bacterial community structure comparing the non-incubated and incubated soils.** Data show the 16S bacterial community structure from soil cores. Incubation had a significant effect on the bacterial community structure, with community structure in soils incubated in the lower environmental chamber (green) or the upper environmental chamber (red) clustering separately from that in non-incubated control soils (blue); *P* < 10^-5^.

**
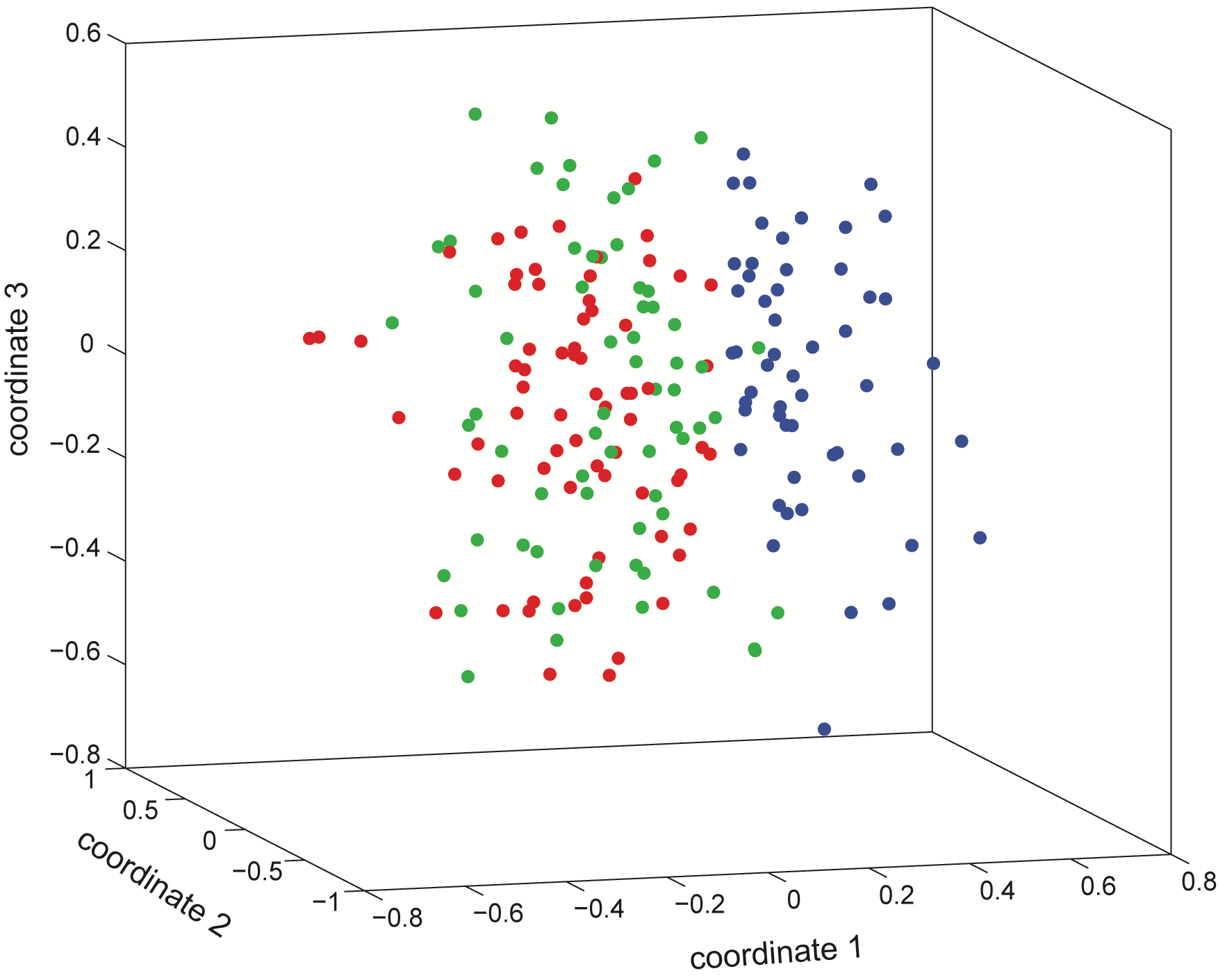
**
